# Evaluation of variations of the glenoid attachment of the inferior glenohumeral ligament by magnetic resonance arthrography

**DOI:** 10.1590/0100-3984.2020.0043

**Published:** 2021

**Authors:** Marcelo Novelino Simão, Maximilian Jokiti Kobayashi, Matheus de Andrade Hernandes, Marcello Henrique Nogueira-Barbosa

**Affiliations:** 1 Central Diagnóstico de Ribeirão Preto (Cedirp), Ribeirão Preto, SP, Brazil.; 2 Hospital das Clínicas da Faculdade de Medicina de Ribeirão Preto da Universidade de São Paulo (HCFMRP-USP), Ribeirão Preto, SP, Brazil.; 3 Digimax Medicina Diagnóstica, Caçador, SC, Brazil.

**Keywords:** Shoulder, Ligaments, articular/anatomy & histology, Magnetic resonance imaging, Ombro, Ligamento glenoumeral inferior, Anatomia, Ressonância magnética

## Abstract

**Objective:**

To evaluate the anatomical variations of the attachment of the inferior glenohumeral ligament (IGHL) to the anterior glenoid rim.

**Materials and Methods:**

This was a retrospective review of 93 magnetic resonance arthrography examinations of the shoulder. Two radiologists, who were blinded to the patient data and were working independently, read the examinations. Interobserver and intraobserver agreement were evaluated. The pattern of IGHL glenoid attachment and its position on the anterior glenoid rim were recorded.

**Results:**

In 50 examinations (53.8%), the glenoid attachment was classified as type I (originating from the labrum), whereas it was classified as type II (originating from the glenoid neck) in 43 (46.2%). The IGHL emerged at the 4 o’clock position in 58 cases (62.4%), at the 3 o’clock position in 14 (15.0%), and at the 5 o’clock position in 21 (22.6%). The rates of interobserver and intraobserver agreement were excellent.

**Conclusion:**

Although type I IGHL glenoid attachment is more common, we found a high prevalence of the type II variation. The IGHL emerged between the 3 o’clock and 5 o’clock positions, most commonly at the 4 o’clock position.

## Abstract (pt)

**Objetivo:**

Avaliar variações anatômicas da inserção do ligamento glenoumeral inferior (LGUI) na margem anterior da glenoide.

**Materiais e Métodos:**

Avaliação retrospectiva de 93 exames de artrorressonância magnética de ombro. Foram realizadas leituras por dois radiologistas para calcular a concordância interobservador e intraobservador. O padrão de inserção glenoidal do LGUI e o seu nível foram registrados.

**Resultados:**

Em 50 exames (53,8%) o LGUI inseria no lábio (tipo 1) e em 43 (46,2%) foi demonstrada uma variação tipo 2. O LGUI emergiu na posição de 4 horas em 58 casos (62,4%), na posição de 3 horas em 14 (15%) e na posição de 5 horas 21 (22,6%). A concordância interobservador e intraobservador para a classificação das variações anatômicas da inserção do LGUI foi excelente.

**Conclusão:**

Embora seja mais comum o LGUI inserir diretamente no lábio anteroinferior, encontramos alta prevalência do LGUI inserindo diretamente na borda óssea da glenoide. O LGUI originou-se entre as posições de 3 e 5 horas, mais frequentemente na posição 4 horas.

## INTRODUCTION

Traumatic shoulder instability is a common condition, with an estimated prevalence of approximately 2% in the general population^(1,2)^. Lesions associated with anterior traumatic shoulder dislocation include bone fractures, such as Hill-Sachs and Bankart lesions, and soft tissue injuries, such as cartilaginous Bankart lesion and its variants. Other shoulder lesions, such as those involving the inferior glenohumeral ligament (IGHL), have also been described in the literature^(3)^.

Humeral avulsion of the glenohumeral ligament (HAGL) reportedly occurs in up to 9.3% of cases of traumatic shoulder dislocation^(4)^. Variations of IGHL lesions described in the literature include avulsion of a bone fragment off the humeral attachment, known as bony HAGL, avulsion of the humeral and glenoid attachments, known as a floating IGHL, and injury associated with posterior dislocation, known as reverse HAGL^(5,6)^. Other possibilities are isolated avulsion of the IGHL off the glenoid rim without lesion of the anterior glenoid labrum, which can be related to detachment but does not involve a tear of the capsular-ligamentous or periosteal complexes^(6,7)^, known as anterior labroligamentous inferior periosteal sleeve avulsion (ALIPSA), or complete capsular-ligamentous avulsion with periosteal tear^(2,3)^, known as glenoid avulsion of the glenohumeral ligament (GAGL), although there is some controversy over these definitions.

While the imaging features of some anatomical variations of the labral aspect or in different capsular, tendinous, and perilabral shoulder attachment patterns, such as the biceps-labral complex, are well-known and have been intensively studied^(8,9)^, variations of the IGHL attachment to the anterior glenoid rim are less common research topics. Most studies in the radiology literature provide only a generic description of the glenoid attachment of the anterior and posterior bands of the IGHL, stating they emerge from the labrum at the middle and lower thirds of the glenoid^(6,10-15)^ or simply from the glenoid rim^(16,17)^, and rarely do we find a more detailed description stating that they originate from the glenoid rim, labrum, and periosteum^(2)^. However, anatomical studies indicate greater variability in the pattern of IGHL glenoid attachment, which can originate from the chondrolabral complex or directly from the glenoid rim^(18,19)^, what Eberly^(19)^ refers to as type I and type II glenoid attachment, respectively. During arthroscopy, it is common to find that the IGHL and the chondrolabral junction cannot be clearly separated at the anteroinferior glenoid rim, a variant known as the labral-ligamentous complex^(20)^.

Several imaging methods can contribute to the diagnosis of lesions associated with traumatic dislocation, including conventional X-ray, conventional magnetic resonance imaging (MRI), magnetic resonance (MR) arthrography, and even ultrasound^(6,7,21,22)^, although MR arthrography is considered the gold standard for the preoperative assessment^(23)^. To our knowledge, there have been no imaging studies aimed at assessing the different patterns of IGHL attachment to the glenoid rim, which could help in the interpretation of this complex anatomy and the associated lesions.

Although an isolated lesion of the IGHL glenoid attachment is rare, it is important to recognize it because, when found in arthroscopies of patients with traumatic shoulder instability, it requires fixation, even if the fibrocartilaginous labrum is intact^(2,24)^. Diagnosing such a lesion with MR arthrography is considered challenging, and some authors argue that the most appropriate arthroscopic approach for diagnosing and assessing the extent of the lesion is the anterior portal^(24)^. A better understanding of the anatomy of the IGHL, combined with the recognition of the different patterns of glenoid attachment, improves the diagnostic capacity of MR arthrography. In addition, details about the lesions obtained during the preoperative assessment with MR arthrography can facilitate the choice of the appropriate surgical techniques and lead to better treatment outcomes.

In light of this, the objectives of this retrospective study were to assess MR arthrography examinations of the shoulder in order to characterize the anatomy of the IGHL attachment to the glenoid rim. We also evaluated the interobserver and intraobserver agreement for the findings.

## MATERIALS AND METHODS

This was a retrospective study carried out in the radiology and diagnostic imaging department of the university hospital of our institution. The study was approved by the research ethics committee of the institution (Reference no. 36029414.7.0000.5440). Because of the retrospective nature of the study, the requirement for written informed consent was waived.

We selected 137 consecutive MR arthrography examinations of the shoulder performed at the university hospital or at a private diagnostic imaging clinic between 2011 and 2013. We applied the following exclusion criteria for MR arthrography examinations of the shoulder: failure to follow the standard protocol (i.e., no acquisition of volumetric sequences); unsatisfactory image quality because of motion or magnetic susceptibility artifacts; poor distension of the joint capsule by the contrast medium; postoperative acquisition; and extensive lesion of the anterior labrum.

On the basis of the study criteria, 44 examinations were excluded. Therefore, the final sample comprised 93 MR arthrography examinations of the shoulder. Of the 93 corresponding patients, 70 (75.3%) were male, with a mean age of 28 ± 9.3 years (range, 14-70 years), and 23 (24.7%) were female, with a mean age of 32 ± 13.2 years (range, 13-65 years). There were 70 patients with a history of traumatic instability, the remaining 23 patients having nothing in their clinical history, medical record, or pre-examination interview that could lead to a suspicion of instability.

### MR arthrography protocols

The MR arthrography examinations involved the acquisition of at least one volumetric sequence in the axial plane (Table 1), which was the only sequence used for assessment in this study. All images were acquired in one of the following MRI scanners:

- A 1.5-T Philips Achieva (Philips Medical Systems, Cleveland, OH, USA), with a SENSE-Shoulder-4 or SENSE-Flex-M coil and acquisition of at least one of the following sequences: three-dimensional (3D) T1-weighted; 3D T1-weighted; 3D T1-weighted VISTA; or proton density- weighted VISTA ISO
- A 1.5-T Signa HDxt (GE Medical Systems, Milwaukee, WI, USA), with a SHLDRPA4 coil and acquisition of a 3D FIESTA sequence
- A 3.0-T Discovery MR750 (GE Medical Systems, Milwaukee, WI, USA), with an HD Shoulder coil and acquisition of a 3D FIESTA sequence

**Table 1:** Parameters of the protocols used in the MR arthrography examinations of the shoulder, by scanner and sequence.

Parameter	Philips Achieva					GE Signa HDxt		GE Discovery MR750
	3D T1	3D T1	3D T1 VISTA	PD VISTA ISO		3D FIESTA		3D FIESTA
Field strength (T)	1.5	1.5	1.5	1.5		1.5		3.0
Repetition time (ms)	8	521-621	373	691		7		7
Time to echo (ms)	4	21	20	35		3		2
Field of view (mm)	180	180	180	180		200		160
Matrix	180 × 180	328 × 306	300 × 257	300 × 257		320 × 320		256 × 256
Slice thickness (mm)	1	1.4	1.4	1.4		0.8		0.8
Interslice gap (mm)	1	0.7	0.7	0.7		0.4		0.4
Bandwidth (Hz/pixel)	192	278	439	258		122		488
Echo train length	180	57	30	65		1		1
Flip angle	8º	90º	90º	90º		35º		35º
Coil		SENSE-Shoulder-4 or SENSE-Flex-M				SHLDRPA4		HD Shoulder

### Data analysis

The images were archived and anonymized, after which they were interpreted by two radiologists, a fellow in musculoskeletal radiology (reader 1) and a senior musculoskeletal radiologist at our institution with four years of experience (reader 2). The two readers, who were working independently, were blinded to the clinical history, signs/symptoms, surgical findings, examination reports, and clinical evolution of the patients. For the assessment of intraobserver agreement, one of the observers (reader 1) re-evaluated the findings six months after the first reading.

**Figure 1:**
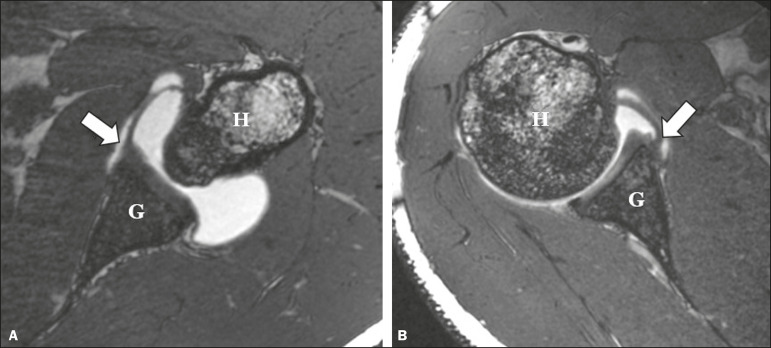
Variations of the AB-IGHL labrum-periosteal glenoid attachment. Axial 3D FIESTA volumetric sequence of the shoulder showing the AB-IGHL (arrow) originating mainly from the labrum—a type I attachment pattern (**A**); and the AB-IGHL (arrow) originating predominantly from the bone margins of the glenoid—a type II attachment pattern (**B**). G, glenoid; H, humerus.

**Figure 2:**
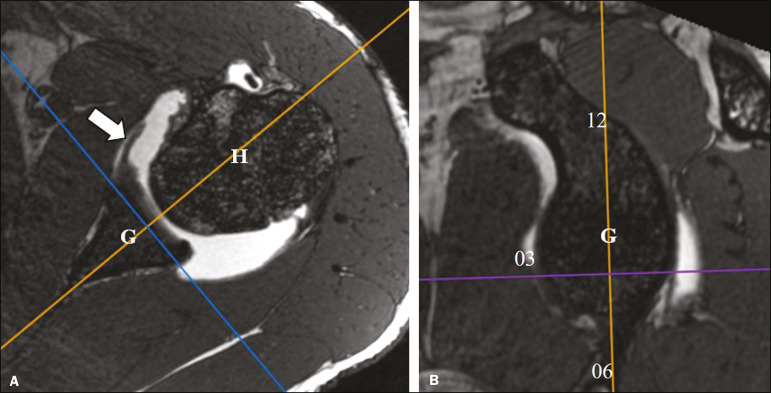
Multiplanar reconstruction tool of OsiriX software on an axial 3D FIESTA volumetric sequence showing the sagittal reconstruction reference lines (**A**); and the sagittal reconstruction plane to identify the level of AB-IGHL attachment to the glenoid rim (arrow) in relation to the 12 o’clock (12), 3 o’clock (03), and 6 o’clock (06) positions (**B**). G, glenoid; H, humerus.

**Figure 3:**
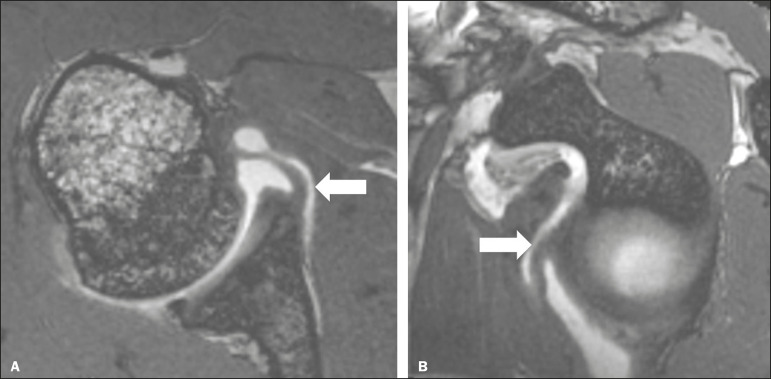
A 30-year-old male patient with no history of traumatic shoulder instability. **A**: Axial 3D FIESTA sequence showing the IGHL emerging directly from the glenoid rim (arrow)—a type II pattern. **B**: Sagittal reconstruction showing the IGHL glenoid attachment at the 3 o’clock position (arrow).

We conducted a retrospective analysis of the cases in order to characterize the following: the glenoid attachment pattern of the anterior band of the IGHL (AB-IGHL), distinguishing between type I, in which the AB-IGHL has a predominantly chondrolabral origin (Figure 1A), and type II, in which it emerges directly from the glenoid rim (Figure 1b); and the AB-IGHL glenoid attachment as a clock position-12 o’clock when it emerged at the upper portion, 3 o’clock when it emerged at the mid-anterior position, and 6 o’clock when it emerged at the lower position-using the multiplanar reconstruction tool of the OsiriX software package (64-bit version; Pixmeo, Geneva, Switzerland) in a three-dimensional volumetric sequence (Figures 2 and 3).

### Statistical analysis

The statistical analysis was performed with the Statistical Analysis System^(24)^, version 9.0 (SAS Institute Inc., Cary, NC, USA). We used the chi-square test to determine whether the types of attachment were associated with the presence or absence of glenohumeral dislocation/instability and labral injury. We used the kappa statistic with a 95% confidence interval (95% CI) for the evaluation of interobserver and intraobserver agreement rates, whereas we used the weighted kappa statistic for the evaluation of the AB-IGHL attachment level. The interpretation of the agreement analysis results was based on the following criteria^(25,26)^: no agreement (κ < 0); poor agreement (κ of 0-0.19); fair agreement (κ of 0.20-0.39); moderate agreement (κ of 0.40-0.59); substantial agreement (κ of 0.60-0.79); and almost perfect agreement (κ of 0.80-1.00).

## RESULTS

### Variation in the AB-IGHL glenoid attachment

The AB-IGHL emerged from the chondrolabral rim (type I pattern) in 50 cases (53.8%) and directly from the glenoid rim (type II pattern) in 43 cases (46.2%). The evaluation of the attachment pattern achieved near-perfect interobserver agreement (κ = 0.91; 95% CI: 0.83-0.99) and intraobserver agreement (κ = 0.87; 95% CI: 0.77-0.97).

After the cases in which the physician request or the patient interview indicated suspicion or complaint of dislocation, instability, or labral injury had been excluded, the sample comprised 23 patients without a clinical history of instability. Of those 23 patients, 15 (65%) had a type I attachment pattern and 8 (35%) had a type II attachment pattern. We found no significant association between the type of attachment pattern and the presence or absence of glenohumeral dislocation/instability and labral injury (*p* = 0.20).

### Level of AB-IGHL attachment to the anterior glenoid rim

The AB-IGHL attached to the glenoid rim at the 4 o’clock position in 58 cases (62.4%), at the 3 o’clock position in 14 (15%), and at the 5 o’clock position in 21 (22.6%). In the assessment of the ligament attachment position, there was substantial interobserver agreement (κ = 0.79; 95% CI: 0.65-0.90) and intraobserver agreement (κ = 0.70; 95% CI: 0.56-0.83).

## DISCUSSION

Several recent studies conducted in Brazil have highlighted the importance of using imaging methods for evaluating the musculoskeletal system^(27-31)^. The anatomy of the glenohumeral ligaments is complex and variable, various patterns having been described in the literature, which makes it difficult to gain a proper understanding of it. Although the IGHL is probably the most widely studied of those ligaments, there is as yet no consensus about its level of origin, size, and most common attachment pattern^(32)^.

The results of the present study confirm those of previous studies showing anatomical variations in the attachment of the AB-IGHL. More than 46% of the cases in our sample had an IGHL attached to the glenoid rim, medial to the fibrocartilage of the glenoid labrum. For the identification of that anatomical variation, the interobserver and intraobserver agreement were almost perfect. Although there are anatomical studies in the literature focusing on the assessment of the different glenoid attachment patterns^(18,19,33)^, descriptions of those aspects on MRI or MR arthrography examinations are less common. In our study, the use of MR arthrography enabled us to analyze a sample larger than those of previous anatomical studies. We were able to identify the IGHL in all 93 of the examinations analyzed, which confirms that MR arthrography is an excellent method for assessing this ligament and that it can be used in routine practice. In addition, the characterization of the anatomy of the AB-IGHL glenoid attachment by a radiologist with experience in the method proved to be reproducible and reliable. Although reports in the literature state that diagnosing isolated lesions of the IGHL glenoid attachment (known as ALIPSA/GAGL lesions) with MR arthrography is a challenge and that arthroscopy is the method of choice in those cases^(24)^, we believe that a better understanding of the IGHL variations and their depiction on MR arthrography can contribute to changing that idea.

Anatomical studies of the variations of the AB-IGHL attachment to the glenoid rim show a predominance of the type I pattern, in which most fibers originate from the chondrolabral rim and some extend to the glenoid rim, with incidence rates of 80-88%, over the type II pattern, in which they originate directly from the bone margin^(18,19)^. In the present study, a predominance of the type I pattern was observed, although the prevalence of the type II pattern, in which the AB-IGHL attaches mainly to the glenoid rim and neck, was also found to be high. A possible reason for that is a selection bias-many patients selected for inclusion in the present study had undergone MR arthrography because of complaints of traumatic shoulder instability, resulting in a specific population that differs from that of asymptomatic people. In an attempt to obtain an unbiased sample, cases in which there was suspicion or a complaint of glenohumeral instability and cases of shoulder dislocation were excluded, after which a new analysis was carried out. In that second analysis, the distribution of the two types was more similar to that reported in previous anatomical studies: type I and type II attachment patterns were observed in 65.2% and 34.8% of the cases, respectively. However, there was no association between the type of glenoid attachment and the presence or absence of glenohumeral instability/dislocation and labral injury.

Because we did not make any arthroscopic correlation, we cannot rule out completely the possibility that some of the patients in our sample had an ALIPSA/GAGL lesion, in which there is avulsion of the IGHL attachment from the glenoid without damage to the anteroinferior labrum^(2,3,6,7)^. Because their findings on imaging examinations are similar, ALIPSA/GAGL lesions can mimic a type II attachment pattern. However, ALIPSA/GAGL lesions are considered quite uncommon, accounting for less than 3% of the labral-ligamentous lesions in patients with a history of shoulder instability^(7)^. Although it is difficult to distinguish between these two conditions on MR arthrography, the presence of a fibrocartilaginous labrum well adhered to the glenoid rim clearly distinguishes a Bankart or Perthes lesion from an ALIPSA/GAGL lesion or a type II attachment of the IGHL to the glenoid rim. The results of this study, in which we found an IGHL glenoid attachment position between 3 o’clock and 5 o’clock, are in agreement with those of anatomical studies of frozen cadavers, which found attachment positions starting at 3 o’clock, with decreasing thickness toward the lower part of the glenoid^(19)^, as well as between 2 o’clock and 5 o’clock, the extent of the attachment being greatest at the 4 o’clock position^(18)^.

Our study has some limitations. First, because it was a retrospective study, the clinical data used were based on the summarized information available on physician requests, electronic medical records, and pre-examination interviews. In addition, there was no standardization of the volume injected into the joint or of the examination protocol. However, a prospective study involving an invasive procedure could raise ethical questions. In addition, although the observers were blinded to the clinical history of patients, findings such as Hill-Sachs and Bankart lesions often made it obvious which cases had a history of traumatic instability. Another limitation is the fact that there is no method of reference or gold standard to confirm the findings of the MR arthrography examinations, which indicates the need for further studies in that area. Furthermore, there could have been a selection bias, given that most of the patients in our sample had a clinical history of shoulder instability, which could affect the usual anatomical aspect of the IGHL-labrum complex. To minimize that limitation, we performed a second analysis, in which cases with a history or signs of instability were excluded. However, as previously mentioned, that analysis was based only on the data available on the physician request and not on a detailed clinical evaluation of the patients. Despite the abovementioned limitations, there are reasons to believe that the results of our study are useful, because they are in agreement with the attachment patterns and levels found in previous anatomical studies, as well as being consistent and having almost perfect interobserver and intraobserver agreement. As in the assessment of other joints, such as the knee menisci^(34)^, identifying these anatomical variations around the anterior glenoid rim on MR arthrography is important for the correct interpretation of the imaging findings.

The treatment of traumatic shoulder instability is often surgical and depends on an accurate preoperative diagnosis and careful planning for each type and degree of injury. Therefore, we can conclude that MR arthrography provides an effective assessment of the anatomy as well as of the different patterns and positions of the AB-IGHL glenoid attachment and that radiologist knowledge about them can contribute to a better understanding of the anatomical variations and of less common lesions of the IGHL-labrum complex associated with anterior shoulder instability, which help achieve correct diagnoses and more appropriate treatment planning.
